# Alterations in the gut microbiota are associated with changes in intestinal antibody production in KK-Ay mice developing type 2 diabetes

**DOI:** 10.3389/fimmu.2026.1766393

**Published:** 2026-06-30

**Authors:** Miho Chikazawa, Ken-Ichiro Minato

**Affiliations:** Department of Applied Biological Chemistry, Faculty of Agriculture, Meijo University, Nagoya, Japan

**Keywords:** dendritic cells, IgA, intestinal immunity, KK-Ay, type 2 diabetes

## Abstract

**Introduction:**

Type 2 diabetes mellitus (T2DM) develops due to multiple factors, including dietary and lifestyle habits and genetic predisposition. In addition, alterations in the immune system have been suggested to be involved in the onset and progression of T2DM. KK-Ay mice, which spontaneously develop T2DM, reportedly exhibit increased IgA antibody production in the intestine and changes in the IgA antibody repertoire. This study investigated changes in the intestinal immune system in T2DM and their underlying mechanisms in KK-Ay mice.

**Methods and results:**

Flow cytometric analysis of immune cells in Peyer’s patches, mesenteric lymph nodes, and the small intestinal lamina propria revealed an increased frequency and activation of immune cells involved in T cell-dependent IgA production pathways. Additionally, KK-Ay mice showed marked changes in the composition of the small intestinal microbiota, along with an increase in bacteria-bound IgA antibodies.

**Conclusion:**

In KK-Ay mice, intestinal antibody production was induced, and changes were also observed in the interaction between the gut microbiota and antibodies, suggesting a reciprocal relationship between these factors. Elucidating the relationship between alterations in the intestinal immune system and disease is expected to contribute to the understanding of disease mechanisms and the development of preventive strategies.

## 1 Introduction

Type 2 DM (T2DM) develops due to impaired insulin secretion by the pancreas combined with insulin resistance in peripheral tissues. In the early stages, the primary clinical features of T2DM are hyperglycemia and hyperinsulinemia. In 2021, the number of individuals with DM worldwide was >500 million, which accounts for >10% of the global adult population (1). As diabetes induces vascular complications that affect multiple organs, such as the kidneys and retina, and results in severe outcomes, including cardiovascular and cerebrovascular diseases, its prevention has become a paramount global challenge (2). The onset of T2DM involves several contributing factors and follows a complex pathogenic process. Therefore, understanding the underlying mechanisms of T2DM development is crucial for establishing effective preventive strategies.

Several mouse models of diabetes have been used in studies on T2DM (3, 4). These include mice that develop obesity and diabetes due to the loss of a single genetic factor, mice that spontaneously develop T2DM, and diet-induced models. Kuo Kondo (KK) mouse is a well-known spontaneous T2DM model that develops obesity, hyperglycemia, and hyperinsulinemia (5). Meanwhile, KK-Ay mice are derived from KK mice by introducing a mutation in the yellow agouti (Ay) gene, resulting in more severe obesity and diabetes symptoms compared with those in KK mice (6). As human T2DM arises from both environmental and genetic factors, multifactorial models more accurately reflect the disease and are valuable experimental systems.

Inflammation is a key factor in the development of T2DM. Patients with T2DM often have increased production of inflammatory mediators, such as tumor necrosis factor alpha (TNF-α), interleukin (IL)-1, IL-6, and IL-18, suggesting a chronic inflammatory state (7). Similarly, in KK-Ay mice, elevated levels of inflammatory cytokines, including TNF-α and IL-6, have been noted, confirming an enhanced inflammatory state (8). Although inflammation can develop secondary to the disease itself, low-grade inflammation is also present prior to T2DM onset and may play an important role in the development of insulin resistance and other diabetes signs and symptoms (9). In obese mice, elevated serum levels of inflammatory mediators such as TNF-α are involved in the development of insulin resistance, indicating that obesity-associated inflammation is another risk factor for diabetes. In T2DM, progression of inflammatory states is closely linked to changes in the intestinal immune system. Alterations in the composition of intestinal immune cells during the development and progression of T2DM may impair intestinal barrier function and disrupt the gut microbiota, potentially contributing to the development of systemic metabolic disorders (10). In the intestine of KK-Ay mice, increased production of mediators, such as histamine and TNF-α, was observed with aging and may exacerbate disease progression (11). Therefore, appropriate regulation of the immune system and suppression of inflammation in T2DM may be potential strategies for disease prevention and mitigation.

Intestinal immunity plays a crucial role in maintaining host homeostasis, with Immunoglobulin A (IgA) antibodies—produced via both T cell-dependent (TD) and T cell-independent (TI) mechanisms—serving as key mediators of this process (12). IgA is the most abundantly produced antibody in mucosal tissues. It is generally considered that the primary antigens of the intestinal immune system are derived from the gut microbiota (13), and IgA contributes to the prevention of infection, protection of tissues, and regulation of the gut microbiota by binding to bacterial antigens (14). In humans with selective IgA deficiency, dysbiosis occurs (15), suggesting that adequate IgA production in the gut is essential for maintaining intestinal homeostasis. Gut inflammation or expansion of pathogenic bacteria may result in the deterioration of the intestinal environment, thereby contributing to the development of chronic and inflammatory diseases (16, 17). Furthermore, intestinal IgA antibodies play a role in suppressing neuroinflammation (18). Collectively the function of the intestinal immune system widely affects various diseases.

In the TD pathway, antigens taken up in Peyer’s patches (PPs) are presented by dendritic cells (DCs) to T cells. Following antigen presentation, T cells are activated and differentiate into follicular helper T (Tfh) cells, which then stimulate B cells and induce IgA class switching (19). The TD pathway is characterized by the production of high-affinity IgA due to antigen-specific selection and formation of plasma cells (PCs) that confer long-term immunity. Meanwhile, the TI pathway is mediated by cytokines such as a proliferation-inducing ligand (APRIL), B cell-activating factor (BAFF), and transforming growth factor-β (TGF-β), which are produced by intestinal epithelial cells, DCs, and other related cells. These cytokines directly stimulate B cells to undergo class switching without requiring T cell involvement (12, 20, 21). Thus, both pathways play important roles in maintaining intestinal homeostasis by producing IgA antibodies with distinct properties through different mechanisms.

Intestinal IgA antibodies attenuate diabetes severity by reducing inflammation in the gut and adipose tissues as well as by counteracting the increase in intestinal permeability. In IgA-deficient mice, high-fat diet (HFD)-induced diabetes symptoms, such as insulin resistance, are exacerbated (22). Additionally, HFD-fed mice showed a reduced number of IgA-positive cells in the lamina propria (LP) (23), suggesting changes in immune cells under dietary stress (22, 24). In recent years, the relationship between the gut microbiota and T2DM has also attracted increasing attention (25, 26). Dysregulation of the gut microbiota has been suggested to be associated with the progression of insulin resistance and impaired glucose tolerance in T2DM, and alterations in microbial diversity have been implicated in these processes. Furthermore, it has been proposed that modulation of the gut microbiota may represent a potential therapeutic strategy for the treatment of T2DM (27). Although the relationship between the development of T2DM and the gut microbiota has been proposed, current knowledge regarding its association with the intestinal immune system remains limited. It has been demonstrated that interactions between intestinal antibodies and the gut microbiota are altered, for example, by an increased proportion of IgA-bound bacteria in the intestine of HFD-fed mice (22). Moreover, it has been suggested that analyzing the diversity and changes in IgA-bound bacteria in saliva and feces of patients with T2DM may be useful for understanding immune responses in T2DM (28); however, the detailed mechanisms underlying these observations remain unclear.

We have previously observed increased antibody production in the small intestinal lumen and alterations in the IgA antibody repertoire in KK-Ay mice (29), suggesting that changes occur in the intestinal immune system in association with T2DM. Currently, the specific alterations in the intestinal immune system of KK-Ay mice, the characteristics of IgA antibodies, and the involvement of the gut microbiota remain unclear. A comprehensive characterization of immune changes in KK-Ay mice should provide novel insights into how genetic factors impact intestinal immunity and how these contribute to disease development. In this study, we aimed to elucidate which alterations in immune cell populations underlie the increased IgA production observed in KK-Ay mice. In addition, we focused on changes in the gut microbiota as a potential driver of altered antibody production and investigated the relationships between these factors.

## 2 Materials and methods

### 2.1 Mice

All experiments were conducted according to the guidelines of the Animal Usage Committee of the Faculty of Agriculture, Meijo University (Aichi, Japan). This study was approved by the ethics committee of Meijo University (Permission No. 2024PE12 and 2024AE13). Six-week-old male C57BL/6JJcl mice and KK-Ay/TaJcl were purchased from Japan CLEA and housed in the animal care facility under controlled temperatures and humidity conditions (24 ± 1 °C, 55 ± 5% humidity) with a 12-h light/dark cycle.

### 2.2 Preparation of tissue samples

Fecal samples from mice were collected at 10 weeks of age. Fecal pellets were harvested 1 h before dissection and weighed, and 100 mg/mL was added to sterile phosphate-buffered saline (PBS) containing 5% fetal bovine serum (FBS) and 0.05% sodium azide. Following incubation for 1 h at 4 °C, the pellets were homogenized using the tip of a 200 µL pipette, particulate debris was removed by centrifugation for 10 min at 13,000 × g, and the supernatants were collected. Fecal samples were used for the measurement of IgA levels. The protein content in feces was determined using Pierce™ BCA Protein Assay Kit (Thermo Fisher Scientific, Cat#23227). Using isoflurane anesthesia, the mice were euthanized at 10 weeks of age. The small intestine was removed and washed, and 5 mL of PBS (pH 7.4) was passed through the intestinal lumen to collect the intestinal fluid. The washout material was then centrifuged at 700 × g for 20 min at 4 °C, and the supernatant was collected and passed through a 70 µm strainer (pluriSelect, Cat# 43-10070-50). The collected intestinal fluid was used for the measurement of protein levels (IgA, IL-6, IL-10, TNF-α, APRIL, and BAFF), NGS-based 16S sequencing, flow cytometric analysis of intestinal bacteria, and isolation of IgA-coated bacteria. Blood samples were allowed to clot for 30 min at room temperature, then serum was obtained by centrifugation at 1,000 × g for 10 min and stored at −80 °C. Serum were used for the measurement of IgA levels.

### 2.3 Preparation of immune cells

To isolate immune cells, PPs were incubated with PBS containing 10 mM EDTA and 5% FBS at 37 °C for 20 min with agitation to remove mucus and epithelial cells. Both PP and MLN cells were minced with scissors and gently triturated using a pestle in PBS, filtered through a 70-μm mesh to remove debris, and centrifuged at 300 × g for 3 min at 4 °C and resuspended in FACS buffer (PBS containing 5% FBS, 1 mM EDTA). For isolation of LP cells, small intestines were transferred into 50-ml conical tubes and shaken for 20 min at 37˚C in PBS containing 10 mM EDTA and 5% FBS. The remaining intestinal tissue was washed and then minced with scissors, transferred into a 50-ml conical tube, and shaken for 40 min at 37˚C in RPMI-1640 (Fujifilm Wako, Cat#189-02025) containing 5% FBS, 1.6 mg/ml of collagenase (Fujifilm Wako, Cat#034-22363), and 0.1 mg/ml of DNAse I (Veritas, Cat#ST-07470). The cell suspension was collected, passed through a 70 µm strainer, centrifuged and resuspended in FACS buffer.

### 2.4 ELISA

Intestinal fluid, fecal, and serum IgA levels were assessed using the ELISA Antibody Pair Kit (STEMCELL Technologies, Cat#01996), according to the manufacturer’s protocol. Cytokines present in the small intestinal fluid were evaluated using the Mouse IL-6 DuoSet ELISA (R&D Systems, Inc., Cat#DY406-05), ELISA MAX™ Standard Set Mouse IL-10 (BioLegend, Cat#431411), Mouse TNF-α DuoSet ELISA (R&D Systems, Inc., Cat#DY410-05), APRIL Do-It-Yourself ELISA (Kingfisher Biotech Inc., Cat#DIY0710M-003), and Mouse BAFF DuoSet ELISA(R&D Systems, Inc., Cat#DY2106-05).

### 2.5 Flow cytometry of immune cells

Immune cells isolated from the PPs, MLNs, and LPs were then blocked with anti-mouse CD16/32 antibody (BioLegend, Cat#101301) dissolved in FACS buffer for 15 min at 4 °C. Following blocking, cells were stained with antibody-fluorophore conjugates for 15 min at 4 °C. Details of the antibodies used are provided in Supplementary Table. Cells were washed and resuspended in FACS buffer, followed by the addition of 7-aminoactinomycin D (7-AAD) (Invitrogen, Cat#00-6993-50) to distinguish live and dead cells, and incubated on ice for 5–10 min. Multiparameter analysis was conducted using a CytoFLEX (BECKMAN COULTER) and analyzed with FlowJo software (Tree Star). For the determination of absolute cell numbers, Flow-Count fluorospheres (Beckman Coulter, Cat#7547053) were added to each sample.

### 2.6 Real-time PCR of immune cells

Immune cells isolated from the PPs and MLNs, and DCs and non-DCs from MLNs were separated using MojoSort Mouse Pan Dendritic Cell Isolation Kit (BioLegend, Cat#480097). The total cell RNA was extracted using TRI Reagent (Molecular Research Center, Cat#TR118) according to the manufacturer’s instructions. Takara PrimeScript ^®^ RT reagent kit (Takara, Cat#RR037A) was employed to synthesize and amplify cDNA from the total RNA. Quantitative real-time PCR was conducted using PowerTrack™ SYBR Green master mix (Applied Biosystems, Cat#A46109) with StepOne Plus system (Life Technologies). Relative mRNA levels were measured by normalizing to the GAPDH transcript. The sequence of the primer sets used in this study was as follows: APRIL, 5′-CGAGTCTGGGACACTGGAAT-3′ and 5′-ATTGTAGGCACGGTCAGGAT-3′; BAFF, 5′-AAACACTGCCCAACAATTCC-3′ and 5′-GTTGCGTGAAATCTGTGCAT-3′; GAPDH, 5′-TGTGTCCGTCGTGGATCTGA-3′ and 5′-CCTGCTTCACCACCTTCTTGA-3′.

### 2.7 NGS 16S sequencing

The intestinal flora in the small intestinal fluid was evaluated by sequencing the 16S ribosomal RNA V3–V4 region by Bioengineering Lab. Co., Ltd. (Kanagawa, Japan). The 16S rRNA gene was amplified using primers 341F and 805R and sequenced on the NextSeq 1000 platform (2 × 300 bp). Reads were quality-filtered using FASTX-Toolkit (v0.0.14) and sickle (v1.33), and merged with FLASH (v1.2.11). Downstream analysis was performed using QIIME2 (v2025.7), where ASVs were generated with DADA2 and taxonomically assigned using the Greengenes database (v13_8).

### 2.8 Flow cytometry of intestinal bacteria

1 ml of the intestinal fluid was passed through a 5 µm strainer (pluriSelect, Cat# 43-10005-50), centrifuged at 8400 × g for 5 min at 4 °C, and resuspended in 1 ml of bacterial FACS buffer (PBS containing 0.1% BSA, 2 mM EDTA). 100 µl of samples were stained with APC-conjugated anti-mouse IgA (Invitrogen, clone mA-6E1) or APC-conjugated rat IgG1 isotype control antibody (Biolegend, clone RTK2071) for 30 min at 4 °C. After washing with PBS, samples were incubated with carboxyfluorescein diacetate (CFDA) solution (Dojindo Laboratories, Cat#BS10) in the dark at 37 °C for 5 min, with propidium iodide (PI) solution (Dojindo Laboratories, Cat#BS10) in the dark at room temperature for 5 min. Multiparameter analysis was conducted using a CytoFLEX (BECKMAN COULTER) and analyzed with FlowJo software (Tree Star).

### 2.9 Isolation of IgA-coated bacteria

Intestinal fluid (500 µL) from KK-Ay mice was resuspended in FACS buffer and incubated with 10 µL of biotinylated anti-mouse IgA antibody (Biolegend, clone RMA-1, Cat#407004) on ice for 30 min. Subsequently, 50 µL of Dynabeads M-270 Streptavidin (Invitrogen, Cat#65305) was added, and the mixture was incubated on ice for an additional 30 min. The fraction bound to the magnetic beads was collected using a DynaMag-Spin Magnet (Invitrogen, Cat#12320D), resuspended in 100 µL PBS, and subjected to 16S rRNA gene sequencing.

### 2.10 Statistical analysis

Statistical analysis was performed using GraphPad Prism 8 (GraphPad Software). Statistical significance was evaluated using the Mann–Whitney U test for two-group comparisons or Tukey’s multiple comparison test following one-way ANOVA for multiple comparisons. All tests were two-tailed, and a *p*-value < 0.05 was considered statistically significant.

## 3 Results

### 3.1 Enhanced intestinal IgA production in 10-week-old KK-Ay mice

**Figure 1:**
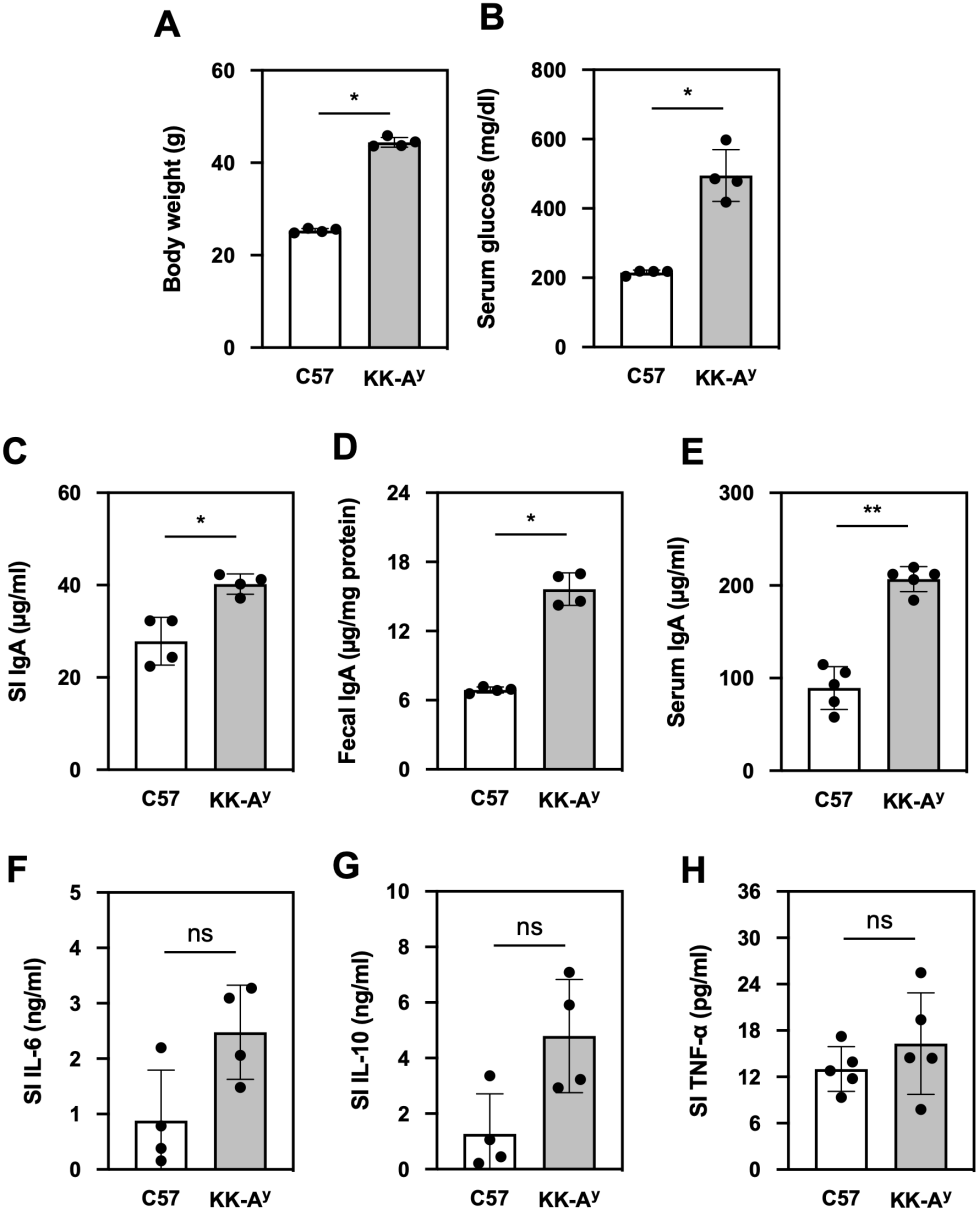
Enhanced IgA production in the intestine of KK-Ay mice. **(A)** Body weight and **(B)** blood glucose levels of C57 and KK-Ay mice. **(C)** Total IgA levels in small intestinal lavage fluid, **(D)** in feces, and **(E)** in serum of mice. Total levels of **(F)** IL-6, **(G)** IL-10, and **(H)** TNF-α in the small intestine. Mice were 10 weeks of age, and there were 4–5 animals per group. Each symbol represents a mouse. Differences were analyzed by the Mann-Whitney U test. *p < 0.05; **p < 0.01; ns, not significant; versus C57. Data represent the mean ± SD where indicated.

To evaluate immune system changes in KK-Ay mice, we assessed alterations in intestinal antibody production. At 10 weeks, KK-Ay mice showed significantly increased body weight (**Figure 1A**) and blood glucose levels (**Figure 1B**) compared with baseline levels, indicating obesity and a diabetic phenotype. Analysis of IgA levels in small intestinal fluid and feces revealed a significant increase in its levels in both the small intestine (**Figure 1C**) and feces (**Figure 1D**), suggesting enhanced antibody production. Furthermore, IgA concentration in the serum was also elevated in KK-Ay mice (**Figure 1E**). Levels of IL-6 and IL-10 showed an increasing trend in the intestinal fluid (**Figure 1F, G**). TNF-α level in the small intestine remained unchanged (**Figure 1H)**.

### 3.2 Increased proportion of IgA-producing cells increases in the small intestine of KK-Ay mice

**Figure 2:**
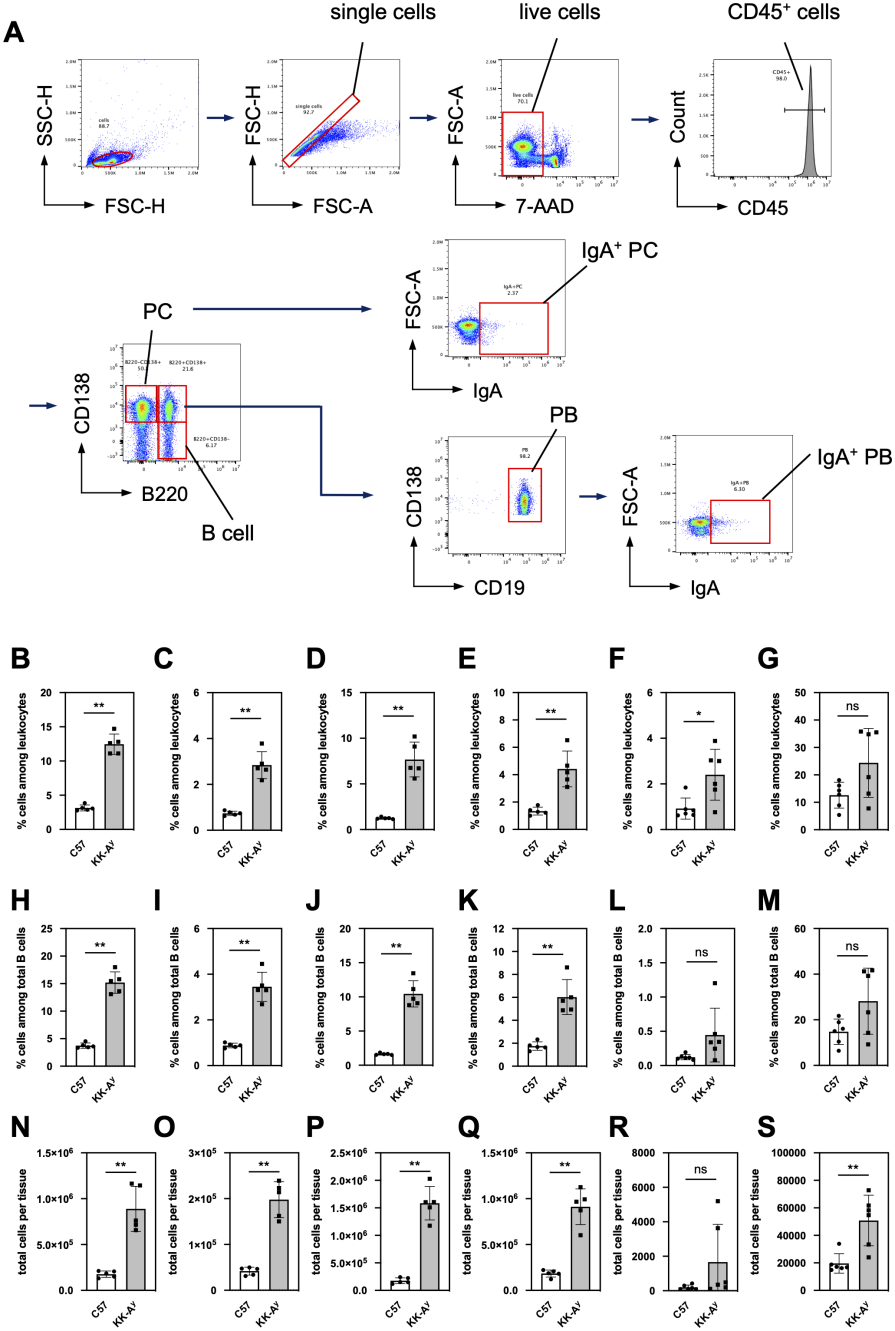
Elevated IgA-producing B cell proportion in PP, MLN, and LP of KK-Ay mice. **(A)** Representative flow cytometry plots of the frequency IgA^+^ PB and PC. PP, MLN, and LP cells from C57 and KK-Ay mice were isolated and stained for analysis by flow cytometry. Cells were pre-gated based on size using FSC-A by SSC-A then doublets were excluded by gating on FSC-A by FSC-H. Live cells were analyzed by gating on 7-AAD-negative cells. Gating on CD45^+^ cells that include leukocytes. PB make up the B220^+^CD138^+^CD19^+^ population and PC cells are found in the B220^-^CD138^+^ population. Both cell populations can be further analyzed for IgA expression. B cells other than PBs and PCs were gated as the population defined by B220^+^CD138^-^. Percentage of **(B)** IgA^+^ PB and **(C)** IgA^+^ PC in PP, **(D)** IgA^+^ PB and **(E)** IgA^+^ PC in MLN, and **(F)** IgA^+^ PB and **(G)** IgA^+^ PC in LP among leukocytes. Percentage of **(H)** IgA^+^ PB and **(I)** IgA^+^ PC in PP, **(J)** IgA^+^ PB and **(K)** IgA^+^ PC in MLN, and **(L)** IgA^+^ PB and **(M)** IgA^+^ PC in LP among total B cells (the sum of PB, PC, and B220^+^CD138^-^ B cells). The total number of **(N)** IgA^+^ PB and **(O)** IgA^+^ PC in PP, **(P)** IgA^+^ PB and **(Q)** IgA^+^ PC in MLN, and **(R)** IgA^+^ PB and **(S)** IgA^+^ PC in LP. Data represent mean ± SD for 5–6 individual mice. Data are representative of three independent experiments. Differences were analyzed by the Mann-Whitney U test. *p < 0.05; **p < 0.01; ns, not significant; versus C57.

As IgA production was found to be increased in the intestinal tract of KK-Ay mice, we hypothesized that some changes might have occurred in immune cells. We therefore investigated B cells, which are responsible for antibody production. B cells were isolated from PP, MLN, and LP, and the proportions of IgA-producing plasmablasts (PBs) and PCs were assessed by flow cytometry (**Figure 2A**). As shown in **Figures 2B–E**, the proportions of IgA^+^ PB (**Figure 2B**) and IgA^+^ PCs (**Figure 2C**) in PP and in MLN (**Figures 2D, E**) in leukocytes were significantly increased. In LP, although the proportion of IgA^+^ PB was increased significantly (**Figure 2F**), IgA^+^ PC remained unchanged (**Figure 2G**). Also, the proportion of IgA-producing PB or PC in total B cell {PB+PC+B cell (B220^+^CD138^-^)} was assessed. The proportions of IgA^+^ PBs and IgA^+^ PCs in PP (**Figure 2H, I**) and in MLN (**Figures 2J, K**) in total B cells were significantly increased. The proportions of IgA^+^ PB and PCs in LP (**Figures 2L, M**) in total B cells remained unchanged. The absolute numbers of IgA^+^ PB and PC in the PP (**Figures 2N, O**) and MLN (**Figures 2P, Q**) were significantly increased. Additionally, no difference was observed in PB of LP (**Figure 2R**), whereas PC was significantly increased (**Figure 2S**). These results are consistent with those presented in **Figure 1**, wherein enhanced IgA production was noted.

### 3.3 Increased proportion of Tfh cells in the small intestine of KK-Ay mice

**Figure 3:**
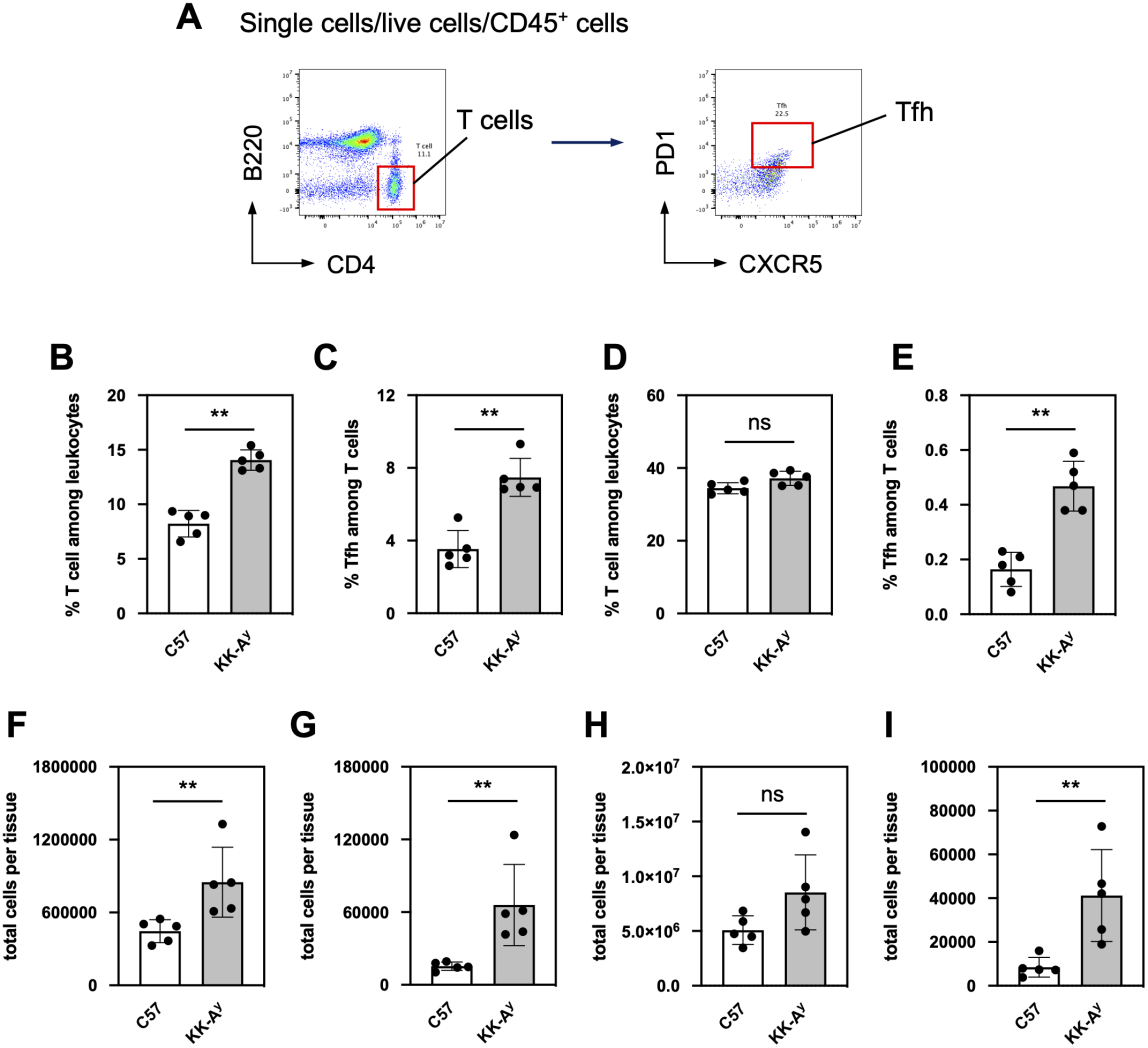
Increased levels of Tfh cells in PP and MLN of KK-Ay mice. **(A)** Representative flow cytometry plots of the frequency of total T cell (CD4^+^B220^−^) and Tfh (CXCR5^+^PD1^+^) levels. Percentage of **(B)** total T cells (among leukocytes) and **(C)** Tfh cells (among T cells) in PP and **(D)** total T cells and **(E)** Tfh cells in MLN. The total number of **(F)** T cells and **(G)** Tfh cells in PP and **(H)** T cells and **(I)** Tfh cells in MLN. Data represent mean ± SD for five individual mice. Data are representative of three independent experiments. Differences were analyzed by the Mann-Whitney U test. **p < 0.01; ns, not significant; versus C57.

We then investigated changes in T cell populations, anticipating effects on T cells responsible for antigen presentation to B cells. We focused our analysis on Tfh cells, which are important for TD IgA production. Leukocytes from PPs and MLNs were evaluated to determine the proportion of total T cells and the fraction of Tfh cells (**Figure 3A**). In PPs, the proportion of T cells (**Figure 3B**) in leukocytes and fraction of Tfh cells among T cells (**Figure 3C**) were significantly elevated. Meanwhile, in MLNs, the proportion of total T cells among leukocytes was unchanged (**Figure 3D**). However, the fraction of Tfh among T cells was significantly increased (**Figure 3E**). In addition, the absolute number of T cells (**Figure 3F**) and Tfh cells (**Figure 3G**) in PP was significantly increased. In MLN, although the absolute number of T cells (**Figure 3H**) did not change, Tfh cells (**Figure 3I**) tended to increase. These finding showed that the proportion of Tfh cells is elevated in both PPs and MLNs.

### 3.4 Alterations in DCs populations in KK-Ay mice

**Figure 4:**
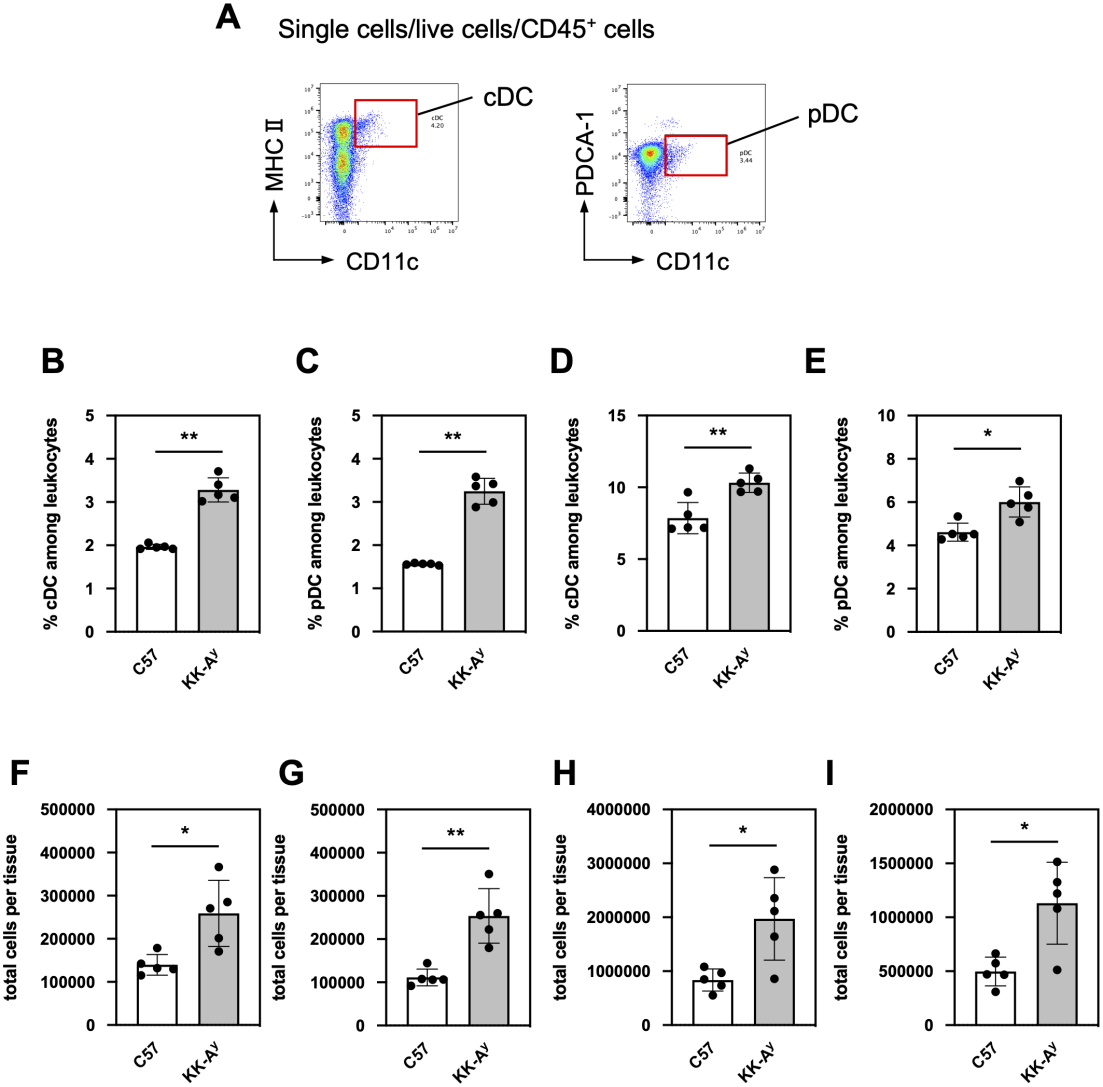
Changes in the proportion of DCs in PP and MLN of KK-Ay mice. **(A)** Representative flow cytometry plots of the frequency of cDC (CD11c^+^ MHCII^+^) and pDC (CD11c^+^ PDCA-1^+^). Percentage of **(B)** cDC, **(C)** pDC in PP, and **(D)** cDC, **(E)** pDC in MLN among leukocytes. The total number of **(F)** cDC, **(G)** pDC in PP, and **(H)** cDC, **(I)** pDC in MLN. Data represent mean ± SD for five individual mice. Data are representative of three independent experiments. Differences were analyzed by the Mann-Whitney U test. *p < 0.05; **p < 0.01; ns, not significant; versus C57.

We then analyzed DCs, which are antigen-presenting cells that initiate the TD pathway upon antigen stimulation. Specifically, the proportion of conventional DCs (cDCs) and plasmacytoid DCs (pDCs) among leukocytes was assessed (**Figure 4A**). In PPs, the proportion of both cDCs (**Figure 4B**) and pDCs (**Figure 4C**) was increased. Also in MLNs, the proportion of cDCs (**Figure 4D**) and pDCs (**Figure 4E**) was significantly increased. Additionally, the absolute numbers of cDC and pDC in the PP (**Figures 4F, G**) and MLN (**Figures 4H, I**) were significantly increased. These results showed that the proportion of DCs is altered in the intestinal immune system of KK-Ay mice, with an overall trend toward increased DC representation in both PPs and MLNs.

### 3.5 Analysis of maturation markers on intestinal cDCs

**Figure 5:**
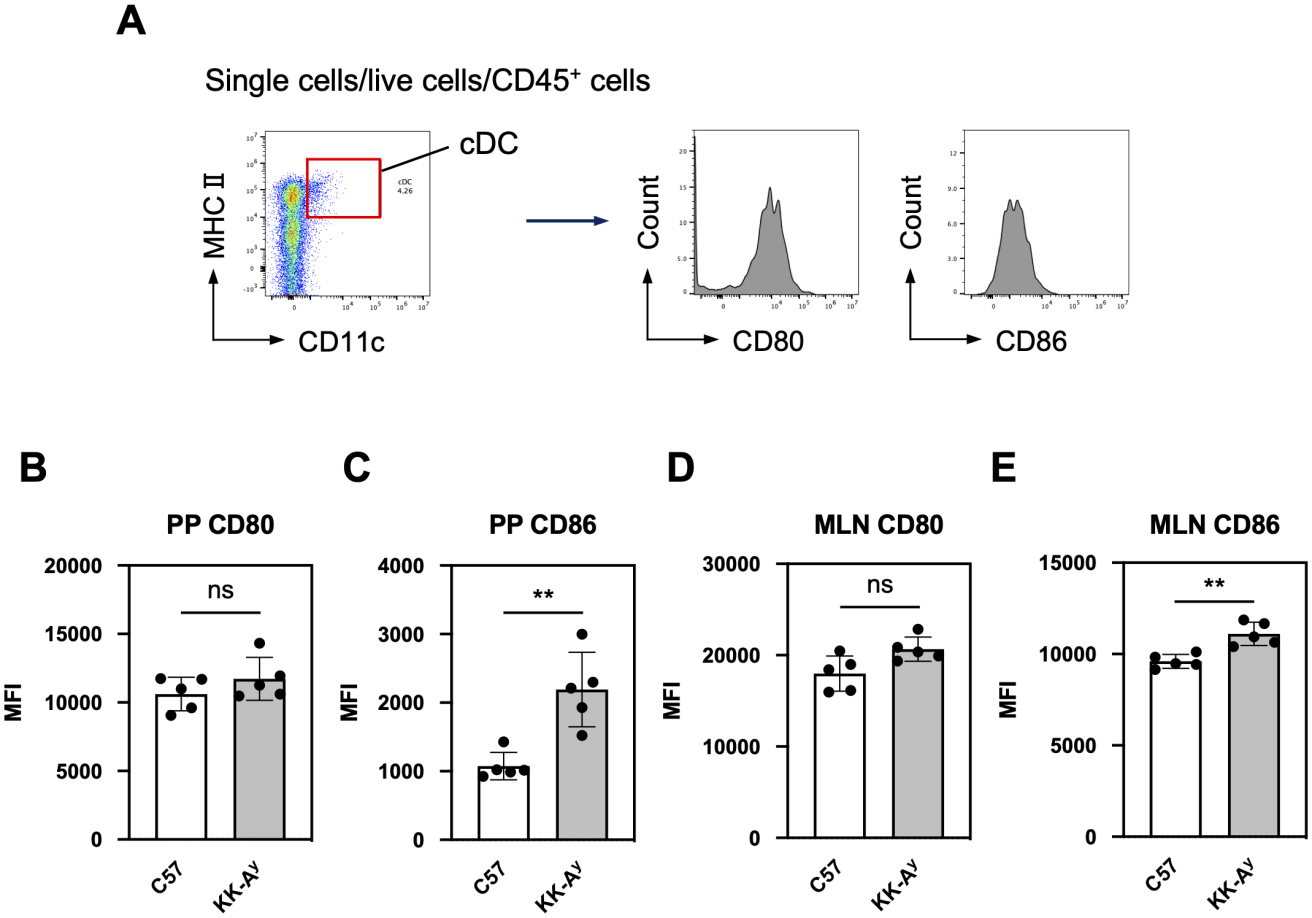
Promotion of cDC maturation in PP and MLN of KK-Ay mice. **(A)** Representative flow cytometry plots of the frequency of cDC (CD11c^+^ MHCII^+^). CD80 and CD86 expression of cDC were compared between groups. MFI of **(B)** CD80 and **(C)** CD86 on cDC in PP, and **(D)** CD80 and **(E)** CD86 on cDC in MLN. Data represent mean ± SD for five individual mice. Data are representative of three independent experiments. Differences were analyzed by the Mann-Whitney U test. **p < 0.01; ns, not significant; versus C57.

Due to the observed increase in cDCs in PPs and MLNs, we assessed the expression of maturation markers on these cells. cDCs were gated from leukocytes isolated from PPs and MLNs, and the mean fluorescence intensity (MFI) of CD80 and CD86 on the gated cells was compared (**Figure 5A**). In PPs, CD80 expression remained unchanged (**Figure 5B**), whereas CD86 expression was elevated (**Figure 5C**). Similarly, in the MLNs, CD80 expression showed no change (**Figure 5D**), whereas CD86 expression was significantly increased (**Figure 5E**).

### 3.6 Analysis of cytokines involved in intestinal TI IgA production

**Figure 6:**
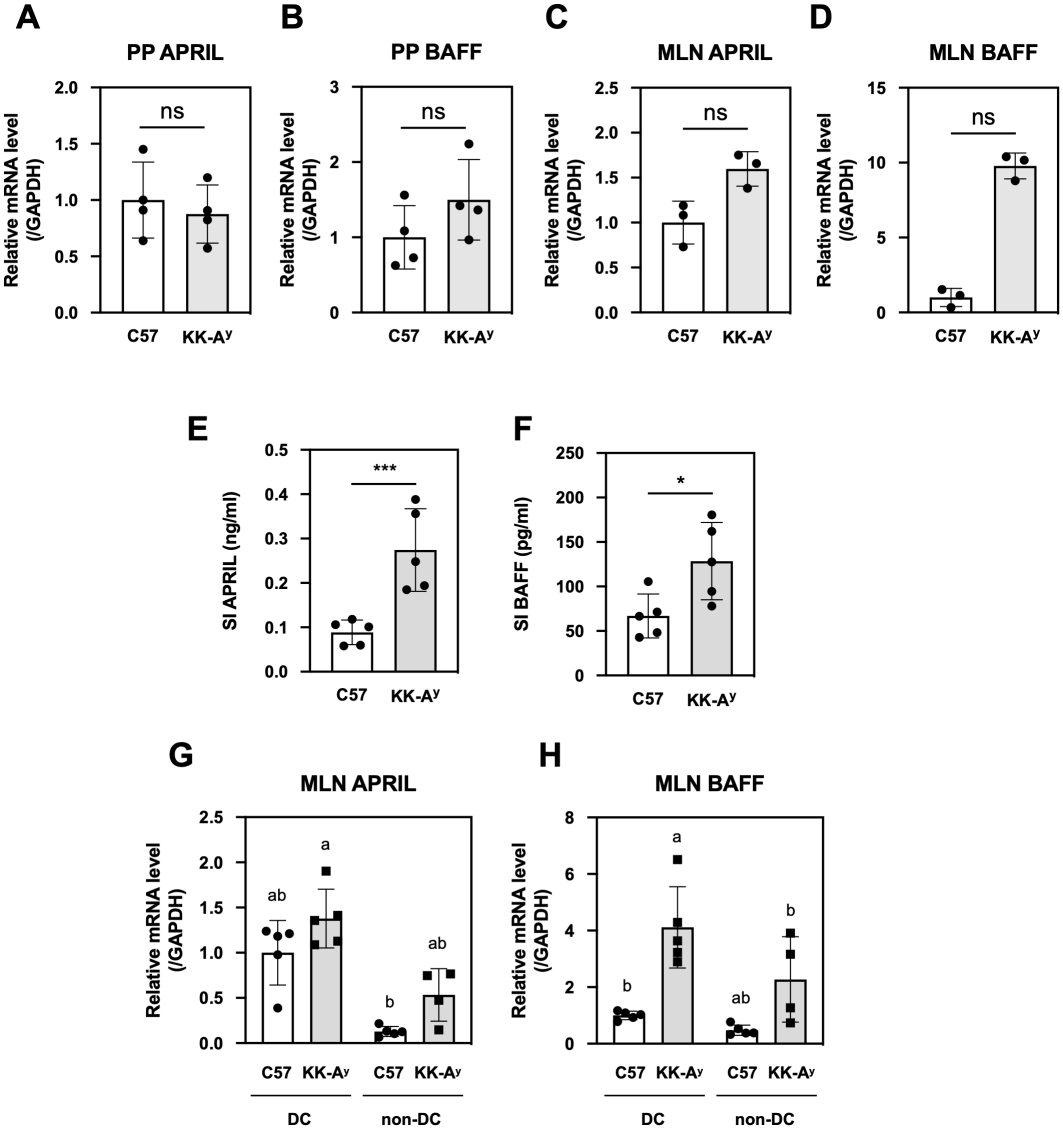
Changes in the expression of TI IgA production-related cytokine genes in mouse PP and MLN. Gene expression of **(A)** APRIL and **(B)** BAFF in PP (n=4/group), and **(C)** APRIL and **(D)** BAFF in MLN (n = 3/group) were evaluated using quantitative PCR. Total protein levels of **(E)** APRIL and **(F)** BAFF in the small intestinal fluids. Real-time PCR analysis of the expression levels of **(G)** APRIL and **(H)** BAFF in DCs and non-DCs from MLN. In figures **(A–D)**, representative data from three independent experiments are shown. In figures **(E–H)**, representative data from two independent experiments are shown. Data are shown as the mean ± SD. In figures **(A–F)**, differences were analyzed by the Mann-Whitney U test. *p < 0.05; ***p < 0.001; ns, not significant. In figures **(G, H)**, differences were analyzed by one-way ANOVA followed by Tukey’s test. Different letters indicate a significant difference (*p* < 0.05).

As DC activation was expected in KK-Ay mice, we evaluated whether the cytokines that promote IgA production via the TI pathway were induced. Cells were collected from PPs and MLNs of 10-week-old mice, and mRNA expression of APRIL and BAFF, which are involved in IgA induction, was assessed by real-time PCR. The expression levels of APRIL and BAFF did not change significantly (**Figures 6A–D**); however, a trend toward increased expression was observed, especially in MLN, with BAFF expression increased by approximately tenfold (**Figure 6D**). To assess whether the production of APRIL and BAFF was increased at the protein level, their levels in small intestinal fluid were evaluated by ELISA. Both APRIL (**Figure 6E**) and BAFF (**Figure 6F**) were significantly elevated in KK-Ay mice. To determine whether these cytokines were produced by DC, further analyses were performed. Cells isolated from MLN were separated into DCs and non-DCs by a magnetic cell separation system, and gene expression was evaluated in each population. As a result, the expression of APRIL showed an increasing trend in both DCs and non-DCs in KK-Ay mice, although the differences were not statistically significant (**Figure 6G**). The BAFF expression was significantly increased in DCs from KK-Ay mice, whereas no significant difference was observed in non-DCs, although an increasing trend was noted (**Figure 6H**). These results suggest that MLN DCs in KK-Ay mice are likely involved in cytokine production; however, the potential contribution of non-DC populations cannot be excluded.

### 3.7 Analysis of alterations in the small intestinal microbiota and IgA-bound bacteria

**Figure 7:**
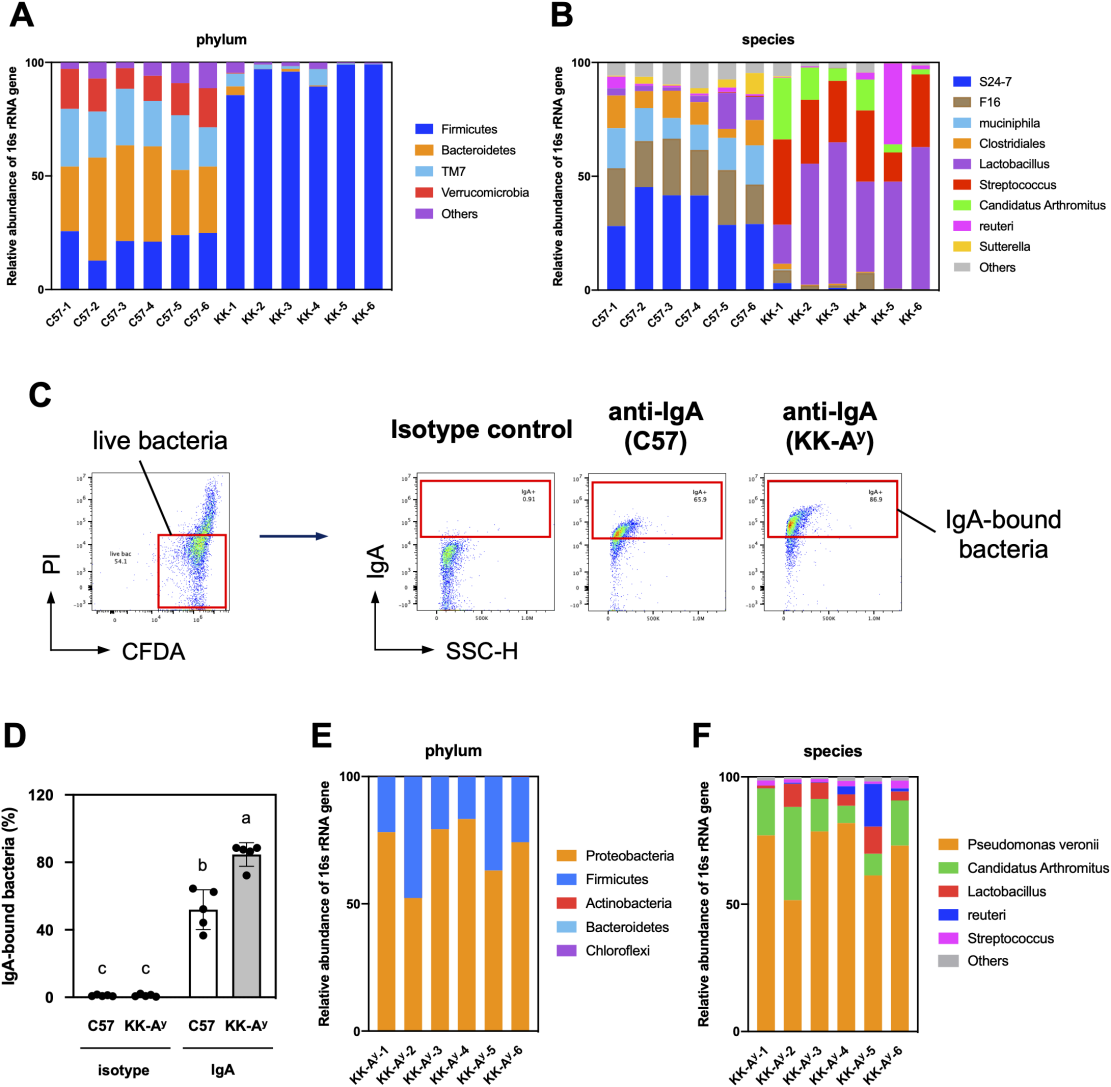
Alterations in the composition of small intestinal microbiota and its binding to IgA in KK-Ay mice. Relative abundances of bacterial communities at the **(A)** phylum and **(B)** species levels in the small intestine of C57 and KK-Ay mice at 10 weeks of age (n = 6/group). Each column represents an individual mouse, and different colors indicate different bacterial genera. **(C)** Representative flow cytometry plots of live (CFDA^+^ PI^-^) small intestinal bacteria from mice stained with anti-mouse IgA antibodies or isotype control antibodies. **(D)** The proportion of IgA-bound bacteria. Relative abundances of bacterial communities at the **(E)** phylum and **(F)** species levels of IgA-bound bacteria in the small intestine of KK-Ay mice at 10 weeks of age (n = 6). Taxa with a maximum relative abundance below 10% **(A)** and 3% **(B, F)** were grouped into a single category and denoted as “Others”. Data are representative of two **(A, B, E, F)** or three **(C, D)** independent experiments. In figure **(D)**, differences were analyzed by one-way ANOVA followed by Tukey’s test. Different letters indicate a significant difference (*p* < 0.05). Data are shown as the mean ± SD.

To analyze the mechanisms underlying the robust IgA production in the intestines of KK-Ay mice, we evaluated the changes in the gut microbiota. First, the overall composition of the small intestinal microbiota was determined via 16S sequencing of luminal wash samples in 10-week-old mice. At the phylum level, Bacteroidetes, TM7, and Verrucomicrobia, which were present at appreciable levels in C57BL/6 mice, were almost absent in KK-Ay mice, with over 94.3 ± 2.3% (mean ± SEM) of the microbiota consisting of Firmicutes (**Figure 7A**). At the genus level, S24–7 of Bacteroidetes, which accounted for 35.7 ± 3.2% (mean ± SEM) of the microbiota in C57BL/6 mice, accounted for 0.7 ± 0.5% (mean ± SEM) in KK-Ay mice (**Figure 7B**). *Lactobacillus* of Firmicutes, which collectively accounted for 5.9 ± 2.4% (mean ± SEM) of the C57BL/6 mice gut, accounted for 46.9 ± 7% (mean ± SEM) of the total microbiota in KK-Ay mice. *Streptococcus* of Firmicutes, which collectively accounted for 0.14 ± 0.067% (mean ± SEM) in C57BL/6 mice, accounted for 28.1 ± 3.4% (mean ± SEM) in KK-Ay mice. *Candidatus Arthromitus* of Firmicutes, which collectively accounted for 0.022 ± 0.0086% (mean ± SEM) in C57BL/6 mice, accounted for 11.0 ± 3.8% (mean ± SEM) in KK-Ay mice. Accordingly, it was revealed that specific bacterial populations were more abundant in KK-Ay mice compared with C57/BL mice.

We then investigated whether IgA produced in the intestines of KK-Ay mice binds to these bacteria. Bacteria were recovered from small intestinal wash samples, live bacteria were gated using CFDA/PI staining, and the proportion of IgA-coated bacteria was evaluated by flow cytometry (**Figure 7C**), revealing a significant increase in IgA-bound bacteria in KK-Ay mice (**Figure 7D**). To determine which bacterial taxa were recognized by IgA, IgA-coated bacteria were separated using magnetic beads and evaluated by 16S sequencing. The IgA-bound fraction contained high proportions of Proteobacteria such as *Pseudomonas veronii* and Firmicutes such as *Candidatus Arthromitus* (**Figures 7E, F**), suggesting that antibodies in KK-Ay mice specifically targeted specific bacterial species.

## 4 Discussion

In this study, we performed a comprehensive evaluation of the changes in the intestinal immune system of KK-Ay mice, specifically on IgA production. Previously, we noted that 18-week-old KK-Ay mice had increased intestinal IgA production along with remarkable changes in the antibody repertoire (29). Accordingly, we hypothesized that antibody production is primarily induced via the TD pathway. Our subsequent assessment confirmed the upregulated activation and increased number of immune cells involved in both TD and TI pathways in KK-Ay mice. Additionally, increased binding of antibodies to intestinal bacteria was observed, suggesting that changes in the small intestinal microbiota may contribute to the induction of antibody production. These findings indicate that the gut microbiota may play a role in modulating intestinal immune changes in the context of diabetes.

To minimize the influence of immune changes secondary to disease symptoms and to focus on changes attributable to the genetic background, we performed our experiments when mice were at 10 weeks of age, which is when disease progression is relatively mild. At this time, KK-Ay mice already exhibited increased body weight and elevated blood glucose, confirming the onset of obesity and diabetes (**Figure 1A, B**). Moreover, IgA levels in the small intestine, feces, and serum were elevated (**Figure 1C, E**). Previous reports also indicated that diabetes symptoms in KK-Ay mice begin to manifest at approximately 10 weeks of age (30, 31), consistent with our findings. As diabetic changes were already present at this stage, all experiments were conducted on 10-week-old mice.

Enhanced inflammation is a well-recognized feature in both patients with diabetes and model mice. Previous studies have reported increases in levels of inflammatory mediators such as TNF-α and histamine in the small intestine of 10-week-old KK-Ay mice, indicating local inflammation (11). In our study, although not statistically significant, a trend toward increased production of IL-6 and IL-10 in the small intestine was observed at the same age (**Figure 1F, G**). This may also be associated with the enhancement of systemic inflammatory status accompanying body weight gain (**Figure 1A**). In contrast, no increase in TNF-α production was detected (**Figure 1H**). TNF-α level was increased in jejunum tissue homogenates in previous report (11), whereas in this study, it was measured in the small intestinal lumen, and this difference in sample type may have influenced the results. These findings suggest that the small intestine of KK-Ay mice exhibits enhanced antibody production, and along with elevated pro- and anti-inflammatory cytokine levels, represent an activated immune state with stimulated immune cells and heightened immune responses. However, at 10 weeks of age, excessive inflammation was not evident.

Due to the observed increase in intestinal antibody production, we focused on alterations in immune cells in the small intestine. In both PPs and MLNs, KK-Ay mice showed a significant increase in the proportion of IgA-producing PBs and PCs among leukocytes (**Figure 2**). This finding is consistent with the elevated IgA levels observed in the intestine and feces (**Figure 1C, D**). PBs are B cells that have been activated by antigen stimulation and are in the process of differentiating into PCs, whereas PCs are fully differentiated B cells. Notably, the proportion of IgA-producing PBs was markedly elevated in both PPs and MLNs (**Figure 2B, D**), indicating that B cells are already activated at 10 weeks of age and likely receiving antigenic stimulation.

We also evaluated the proportion of Tfh cells, which are important for antigen presentation and play a role in the TD pathway. In both PPs and MLNs, the proportion of Tfh cells among T cells was elevated (**Figure 3C, E**). Tfh differentiation is induced not only by antigenic stimulation but also by IL-6 produced by DCs as well as chronic intestinal inflammation such as in inflammatory bowel disease (32, 33). Chronic inflammation associated with diabetes (7) and the observed increase in IL-6 production in the small intestine (**Figure 1F**) are also consistent with these findings. Collectively, these results indicate that various stimuli in the KK-Ay intestine promote Tfh differentiation.

DCs play an important role in IgA production by presenting antigens to T cells, which initiate the TD pathway, and by producing cytokines in response to microbial stimuli, which induce B cell class switching in a TI manner (34, 35). Meanwhile, cDCs are mostly involved in antigen presentation and T cell activation, whereas pDCs contribute to regulatory T cell induction and antiviral responses. In this study, the proportion of cDCs was significantly elevated in both PPs and MLNs (**Figure 4B, D**). It is aligned with our hypothesis that there is active TD IgA production, consistent with the increases in the number of IgA-producing PBs and PCs (**Figure 2**) and Tfh cells (**Figure 3**). Upon antigen stimulation, there is usually an increase in the expression of maturation markers on cDCs (36). In KK-Ay mice, we noted elevated expression of CD86 on PP and MLN cDCs (**Figure 5C, E**), indicating that cDCs in both PPs and MLNs are activated and in a mature state. However, in the present study, antigen-presenting capacity was not directly evaluated; therefore, more detailed analyses of T cell responses will be necessary in future studies.

DCs promote IgA production not only through antigen presentation but also via cytokine secretion. Transmembrane activator and CAML interactor (TACI) is an immune receptor that is primarily expressed on B cells. TACI is a receptor for the TNF-family ligands APRIL and BAFF (21). Upon activation by these ligands, TACI induces class switch recombination in B cells, leading to their differentiation into IgA-producing cells. The protein levels of APRIL and BAFF in the intestinal fluid were elevated (**Figure 6E, F**). Accordingly, it was speculated that the TI IgA production pathway might also be induced through cytokine production in KK-Ay mice. pDCs in MLNs have been reported to induce TI IgA production via APRIL and BAFF (12), and in the present study, both the proportion and number of pDCs were increased in the MLNs of KK-Ay mice (**Figure 4E, I**). BAFF is produced not only by DCs but also by monocytes, macrophages, and neutrophils, and its expression is induced by cytokine stimulation or microbial components (37). Our study suggested that, in addition to DCs, other immune cells may also contribute to BAFF production (**Figure 6H**). Although the identification of non-DC cell types involved in BAFF production will be necessary in future studies, DCs are likely to contribute to TI IgA production through BAFF production.

BAFF production can be induced by type I interferons, interferon-γ, TGF-β, IL-10, and granulocyte-colony stimulating factor (37). Thus, the observed increase in the production of IL-10 (**Figure 1G**) in KK-Ay mice may contribute to BAFF induction. Conversely, BAFF stimulation strongly induces IL-10 production by B cells, indicating a potential positive feedback loop between IL-10 and BAFF expression (38). While BAFF stimulation of B cells promotes proliferation and maturation, it may also be involved in the development of systemic lupus erythematosus and other autoimmune diseases (38). As autoimmune-like responses may play a role in the onset and progression of T2DM (39), future studies should investigate whether similar autoimmune-like phenomena occur in KK-Ay mice.

Based on our immune cell analyses, the proportion of cells involved in the TD pathway was markedly increased in 10-week-old KK-Ay mice, suggesting robust induction of the antibody production. This also suggests that levels of novel antigens in the gut are elevated and stimulate specific antibody production via the TD pathway. As the gut microbiota serve as major antigens for intestinal IgA antibodies (13), changes in its composition may increase levels of bacterial antigens and trigger the TD response. Therefore, we also analyzed the composition of the small intestinal microbiota, revealing that there were distinct differences between control and KK-Ay mice (**Figure 7A, B**). Uchikawa et al. reported that, at the phylum level in feces, KK-Ay mice had a higher abundance of Firmicutes compared with control mice (40). This is consistent with our observation that KK-Ay mice had a high proportion of Firmicutes in the small intestine lumen, although differences between feces and the small intestine must be noted. In contrast, in Tsumura Suzuki Obese Diabetes mice, which also spontaneously develop diabetes, the small intestinal microbiota was not significantly different to that with control mice at the phylum level; however, at 5 weeks of age, *Lactobacillus* (Firmicutes) was more abundant than *Candidatus Arthromitus* (Firmicutes), which was prevalent in control mice (41). In contrast, our study revealed that *Candidatus Arthromitus*, which was barely detectable in control mice, constituted accounted for 11.0 ± 3.8% (mean ± SEM) of the microbiota in KK-Ay mice (**Figure 7B**). Although a detailed comparison would require creating different model mice under identical conditions, these results indicate that even spontaneously diabetic mouse models do not necessarily display identical gut microbiota patterns. Determining whether these differences in the gut microbiota develop from variations in the disease development process and identifying the underlying factors may help clarify the relationship between microbiota alterations and diabetes onset. As it is hypothesized that an abnormal gut flora can induce the development of diabetes (42), the role of the gut microbiota in diabetes should be addressed by future studies.

There are several reports on the changes in the gut microbiota induced by HFD feeding in mice. HFD intake decreases the *Bacteroidetes* population and increases the *Firmicutes* and *Proteobacteria* in fecal microbiota (43, 44), which was consistently observed across several studies (45). Although these findings reflect fecal rather than small intestinal bacteria, similar alterations are expected in KK-Ay mice during diabetes development. Previously (29), the antibody repertoires of HFD-fed mice and KK-Ay mice were markedly different, indicating that the specificities of antibodies and the antigens that induce antibodies differ between these models. Although some alterations in the gut microbiota of KK-Ay and HFD mice are similar, microbiota alterations alone are unlikely to fully account for changes in antibody repertoires. However, on the other hand, an increase in the proportion of IgA-coated bacteria in the small intestine has been observed in both KK-Ay mice (**Figure 7D**) and HFD-fed mice (22), suggesting that a common immune response to the gut microbiota may be occurring. Further comparisons, including evaluations of antibody repertoires and gut microbiota in KK-Ay mice fed with HFD, may provide deeper insights into the distinct repertoire changes observed in KK-Ay versus diet-induced models.

S24-7 (*Muribaculaceae*), which is a member of the Bacteroidetes phylum, is abundant in the murine gut and can metabolize carbohydrates (46). In the small intestine of control mice, S24–7 accounts for 35.7 ± 3.2% (mean ± SEM) of the microbiota, whereas its abundance in KK-Ay mice accounts for 0.7 ± 0.5% (mean ± SEM) (**Figure 7B**). S24–7 can metabolize extracellular polysaccharides from *S. salivarius* to produce short-chain fatty acids (SCFAs) (47) as well as induce TI IgA production via SCFA production (48). The low population of S24–7 in KK-Ay mice may result in insufficient TI IgA induction, potentially impairing homeostatic IgA production and contributing to disease onset. Furthermore, HFD feeding reportedly reduces S24–7 populations (49), and a decrease in S24–7 may be a common feature in T2DM. As S24–7 may be protective against type 1 diabetes (50), a decreased in abundance may contribute to diabetes development, underscoring the need for further investigation into its *in vivo* effects.

*Pseudomonas veronii* accounted for 70.6 ± 4.8% (mean ± SEM) of the IgA-bound bacteria in KK-Ay mice (**Figure 7F**), suggesting that it may be a major target of IgA antibodies produced in these mice. *Pseudomonas veronii*, which is usually found in soil or water, has also been detected in the intestines of malaria-infected mice (51). The biological role of *Pseudomonas veronii* in the gut remains unclear. As IgA-bound bacteria can reportedly induce colitis (13), its effects on intestinal immunity need further investigation. It is also possible that antibody production against *Pseudomonas veronii* limits its overgrowth, which may explain its low abundance in the small intestine.

IgA antibodies binding to *Candidatus Arthromitus*, known as segmented filamentous bacteria (52), were observed in the small intestine of KK-Ay mice (**Figure 7F**). This bacterium is highly immunostimulatory: i.e., colonization induces strong IgA production and Th17 differentiation (53). *Candidatus Arthromitus* poorly induces TI responses but triggers TD IgA production (54), leading to the induction of antigen-specific IgA (55), which may be attributed to unique antigenic structures on its cell surface. The increased proportion of this bacterium in KK-Ay mice (**Figure 7B**), together with these previous reports, suggests that its expansion may be involved in the activation of TD IgA production. To demonstrate this, it is necessary to determine whether the antibodies binding to this bacterium are also present in normal mice or are specifically induced in KK-Ay mice. Additionally, as *Candidatus Arthromitus* can promote IL-17 production (56), its overgrowth could potentially contribute to intestinal inflammation. Therefore, the increased IgA binding to *Candidatus Arthromitus* may suppress its activity and contribute to the maintenance of mucosal homeostasis. Future studies that evaluate the specific immune responses against *Candidatus Arthromitus* are important for understanding host–microbe interactions. Moreover, as both *Pseudomonas veronii* and *Candidatus Arthromitus* accounted for most of the IgA-bound bacteria (**Figure 7F**), they may share common antigenic structures that are recognized by the same antibodies, warranting further assessments of antibody specificity and cross-reactivity.

In summary, activation of immune cells was observed in the intestine of KK-Ay mice, suggesting that both TD and TI IgA responses are enhanced. Alterations in the gut microbiota, together with the presence of bacteria-specific IgA, suggest that antigen-specific immune responses may influence changes in the intestinal antibody repertoire in KK-Ay mice. However, further studies are required to clarify this causal relationship, including direct evidence demonstrating that microbiota alterations drive immune responses. In addition, it remains necessary to determine whether IgA antibodies binding to bacteria are produced via the TD or TI pathways, and whether they are specifically induced in KK-Ay mice in response to microbiota changes. The affinity and specificity of these antibodies toward intestinal bacteria also remain unclear, as does whether they exert beneficial effects, such as suppressing the growth of harmful bacteria. It is also possible that IgA induced in a compensatory manner in response to bacterial expansion may lead to unfavorable outcomes for the host. Future studies focusing on the mechanisms of antibody production and functional analyses of bacteria-binding antibodies are expected to provide detailed insights into how alterations in intestinal antibody responses contribute to the pathogenesis of diabetes.

## Data Availability

The datasets presented in this study can be found in online repositories. The names of the repository/repositories and accession number(s) can be found below: https://www.ddbj.nig.ac.jp/, DRR958296-DRR958313.
